# New technology can benefit established middle ear implant users: Samba 2 vs previous models of audio processors for Vibrant Soundbridge

**DOI:** 10.1007/s00405-022-07741-9

**Published:** 2022-11-28

**Authors:** Anna Ratuszniak, Artur Lorens, Anita Obrycka, Justyna Witkowska, Henryk Skarzynski, Piotr Henryk Skarzynski

**Affiliations:** 1 Otorhinolaryngosurgery Clinic, World Hearing Center, Institute of Physiology and Pathology of Hearing, Mochnackiego 10 Str., 02-042 Warsaw, Poland; 2 Implant and Auditory Perception Department, World Hearing Center, Institute of Physiology and Pathology of Hearing, Mochnackiego 10 Str., 02-042 Warsaw, Poland; 3 Department of Teleaudiology and Hearing Screening, World Hearing Center, Institute of Physiology and Pathology of Hearing, Mochnackiego 10 Str., 02-042 Warsaw, Poland; 4 Heart Failure and Cardiac Rehabilitation Department, Medical University of Warsaw, 8 Kondratowicza Str., 03-242 Warsaw, Poland; 5 Institute of Sensory Organs, Mokra 1 Str., 05-830 Kajetany, Poland

**Keywords:** Middle ear implant, Vibrant Soundbridge, Semi-implantable hearing aid, Audio processor, Partial deafness

## Abstract

**Introduction:**

The Vibrant Soundbridge (VSB) is a semi-implantable hearing aid for patients with various types of hearing loss and has been available for over 25 years. Recently, new audio processors with advanced signal processing, noise reduction, and multi-microphone technology have appeared. The aim of this study is to compare the benefits of using the newest Samba 2 processor to the previous generation processors in a group of experienced VSB users.

**Methods:**

There were 22 experienced VSB users (mean time of using VSB was 9 years, SD = 2) who had their processor (D404 or Amadé) upgraded to the newest model (Samba 2). The mean age of the subjects was 56 years (SD = 20). Assessments were made by free-field audiometry, speech reception in quiet and noise, and Patient-Reported Outcome Measures (PROMs).

**Results:**

Hearing tests in free field showed statistically significant improvements in hearing sensitivity and speech discrimination in quiet and noise with the Samba 2 audio processor compared to the earlier technology. PROMs confirmed the benefits of using the newest audio processor and there was more satisfaction in terms of usability.

**Conclusions:**

Access to modern technology for VSB patients provides measurable benefits.

## Introduction

The Vibrant Soundbridge (VSB) is a partially implanted middle ear device (Med-El, Innsbruck, Austria) which was introduced in 1996 [1, 2]. It is intended for patients with acquired and congenital moderate to severe sensorineural, conductive, or mixed hearing loss who have contraindications to, or limitations with, the use of conventional hearing aids [3–5]. The approach of having the FMT attached to the long process of the incus was introduced to treat patients with SNHL. The newer coupling techniques using various types of couplers have been developed for treating conductive and mixed hearing loss. Currently, the FMT can also be placed on the round window, using direct or indirect coupling [6–8]. The 25-year history of this solution has led to multiple reports of its safety, effectiveness, and improvement in the quality of life of patients [9–12]. During this time there have also been new developments in medical technology. In terms of middle ear implants, there have been several generations of audio processors (APs) with new functions. The first VSB was the D404 (transferred to Med-El in 2003), the Amadé processor introduced in 2009, and the Samba processor in 2015. The latest VSB processor is the Samba 2, launched in 2020. New functions include advanced signal processing, noise reduction, multi-microphone technology, speech tracking, and remote control. Signal processing and functionality have improved over the generations [13]. The multi-microphone technique (on which we focus here) has been used for several years in both hearing aids and various types of implants [14–17]. It was presumed the technique would bring measurable benefits to our study group, although the improvements are difficult to measure objectively because of the built-in adaptive circuitry: measurements would need to simulate a rapidly changing acoustic scene to reproduce the conditions under which adaptive microphones operate. An easier alternative is to assess the benefits of using this technology by using Patient-Reported Outcome Measures (PROMs). The combination of both objective and subjective measures allows a reliable assessment to be made of the device’s benefits—understood as the reduction in the user’s limitations caused by hearing loss.

Thanks to the compatibility of newer APs with older implants, access to modern technology is even possible for long-term users. Patients can gain in two areas: they receive a new device that is more reliable than the old one, and they gain access to modern technology that was unavailable in their legacy processor. To the authors' knowledge, there has been only one previous study on upgrading the VSB technology—from Samba to Samba 2 [18].

The aim of this study was to assess the benefits to users from upgrading their previous generation processor to Samba 2 in terms of hearing sensitivity and speech discrimination outcomes as well as in terms of PROMs.

## Methods

### Subjects

There were 22 VSB users who were enrolled in this study (13 female, 9 male). The subjects were implanted between 2009 and 2015 with a VSB (either a VORP 502 or VORP 503) and were provided with a D404 or Amadé audio processor. The mean age of the subjects was 56 years (SD = 20, range 14–79 years) at the time of the upgrade visit. The mean time of VSB use was 9 years (SD = 2, range 5–12 years). Details are given in Table 1.

**Table 1:** Demographic and audiometric details

Subject no.	Gender	Time of VSB use (years)	Age at SAMBA 2 fitting (years)	Implanted ear	PTA4 (AC) [dB HL]	PTA4 (BC) [dB HL]	Type of hearing loss in the upgraded ear
1	M	12	28	R	76.3	18.8	CHL
2	M	7	79	R	53.8	48.8	SNHL
3	M	9	59	R	48.8	43.8	SNHL
4	M	12	74	L	62.5	56.3	SNHL
5	F	7	28	L	68.8	6.3	CHL
6	F	10	65	R	62.5	11.3	CHL
7	F	9	63	L	73.8	31.3	MHL
8	F	8	26	L	83.8	28.8	MHL
9	F	9	35	R	71.3	65.0	SNHL
10	F	12	66	R	66.3	60.0	SNHL
11	F	10	23	R	47.5	6.3	CHL
12	M	11	73	R	58.8	46.3	SNHL
13	F	11	63	L	77.5	32.5	MHL
14	F	9	57	L	42.5	12.5	CHL
15	M	11	59	R	87.5	53.8	MHL
16	M	12	66	R	98.8	41.3	MHL
17	M	7	72	R	62.5	52.5	SNHL
18	F	6	76	L	88.8	56.3	MHL
19	F	10	66	L	70.0	37.5	MHL
20	F	7	14	R	90.0	40.0	MHL
21	M	9	73	L	70.0	37.5	MHL
22	F	5	68	R	62.5	51.3	SNHL

*F* female, *M* male, *R* right, *L* left, *AC* air conduction, *BC* bone conduction, *PTA4* pure tone average for 0.5, 1, 2, 4 kHz, *CHL* conductive hearing loss, *MHL* mixed hearing loss, *SNHL* sensorineural hearing loss

### Audio processors

In 6 cases a D404 audio processor was used and in 16 cases Amadé, all with the omni-directional microphone switched on in the universal program setting. They were all fitted at standard follow-up visits. All processors were replaced with Samba 2 (in 12 cases on the right, in 10 on the left). The Samba 2 AP was individually fitted to the patient based on a Vibrogram (in situ measurement of hearing thresholds) with SYMFIT, version 8.0.1, following a first fitting session using DSL version 5 for Samba 2. Samba 2 was set with directionality, surround sound, and voice tracking on in the universal program. An overview of the differences between processors of different generations is shown in Table 2.

**Table 2:** Comparison of the audio processors

	D404	Amadé	Samba 2
Frequency shaping			
Digital signal processing	No	Yes	Yes
Adjustable channels	8	16	18
Compression channels	4	8	18
Pre-processing			
Wind-noise reduction	No	Yes	Yes
Speech in noise management	Yes	Yes	Yes
Sound smoothing	No	Yes	Yes
	Yes	Yes	Yes
Microphone options			
Directional microphone	No	Fixed directionality	Automatic adaptive directionality
Environmental sound classification	No	No	Yes (Intelligent Sound Adapter 2.0 with classifier)
Automatic microphone setting	No	No	Yes
Speech tracking	No	No	Yes
Audio frequency range	250–8000 Hz	250–8000 Hz	250–8000 Hz

### Outcome measures

*Pure-tone audiometry* Pure-tone audiometry was performed in an anechoic chamber with an Otometrics Madsen Itera II diagnostic audiometer. Air conduction thresholds were measured with TDH39 on-ear headphones at 0.125, 0.25, 0.5, 1, 2, 4, and 8 kHz; bone conduction was measured using a B-71 calibrated bone transducer at 0.25, 0.5, 1, 2, and 4 kHz. If there was no response to sound during the test, the maximum value available in the audiometer for the given stimulus was taken to be the threshold value.

*Sound field tests* Sound field thresholds were tested using warble tones from a loudspeaker 1 m in front of the subject at 0.25, 0.5, 1, 2, 4, and 6 kHz under unaided and aided conditions (in both the old and new APs). Word recognition score in quiet (WRS) was measured using the Polish Monosyllabic Word Test at 65 dB SPL from the front under unaided and aided conditions (in the old and new AP). The speech perception threshold in noise, at which the subject can correctly repeat 50% of the words in a presented sentence (SRT_50_), was measured with the Polish Matrix Sentence Test under two conditions: with signal and noise presented from the front (S0-N0); and with signal presented from the front and noise from the back (S0-N180). The noise level was fixed at 65 dB SPL, and the speech presentation level was adapted according to the measuring protocol; measurements were conducted under the aided condition (in old and new AP).

*Patient-Reported Outcome Measures (PROMs)* Subjective benefit was determined using the Speech, Spatial, and Qualities of Hearing Scale Questionnaire (SSQ12), and the Audio Processor Satisfaction Questionnaire (APSQ) for both aided conditions (Samba 2 and the previous generation AP). The Speech, Spatial, and Qualities of Hearing Scale Questionnaire (SSQ12) consists of 12 items divided into 3 dimensions (speech hearing, spatial hearing, and qualities) and uses a scale from 0 to 10 [19]. Higher scores indicate less disability. The APSQ consists of 15 items divided into 3 dimensions (comfort, social life, and usability) and is scored between 0 and 10 [20].

Higher scores indicate more satisfaction with the AP. The questionnaires were completed twice before the upgrade and after 5–10 weeks of new AP use, either electronically or in hard copy sent by post.

### Statistical analyses

A Student’s *t* test was used to make pair-wise comparisons of patients’ outcomes between the old AP and the new AP in terms of sound field thresholds, speech outcomes (word recognition scores in quiet and speech discrimination in noise), and PROMs. The hypothesis of normal distribution of the data was evaluated using a Shapiro–Wilk test. The relationships between speech outcomes and PROM results were analyzed by means of Pearson correlations. The level of significance was set at *α* = 0.05.

The study was approved by the Institutional Review Board of the Institute of Physiology and Pathology of Hearing (KB.IFPS: 3/2022) and was conducted in accordance with the Helsinki Declaration.

## Results

### Pure-tone audiometry

The unaided mean AC PTA4 in the implanted ear (average air conduction thresholds for 0.5, 1, 2, and 4 kHz) was 69.3 dB (SD = 14.7). The BC PTA4 (bone conduction for the same frequencies) was 38.1 dB (SD = 17.8). Audiometric details for individual patients are shown in Table 1.

### Sound field tests

Sound field thresholds improved considerably with both devices over unaided conditions. The mean unaided free-field PTA4 was 69.9 dB (SD = 12.6), whereas with Samba 2 it was 38.1 dB (SD = 7.4) and with the old AP it was 42.1 dB (SD = 11.6). The 4 dB improvement with the new AP compared to the old was significant (*t*(21) = 2.47; *p* = 0.022).

**Fig. 1:**
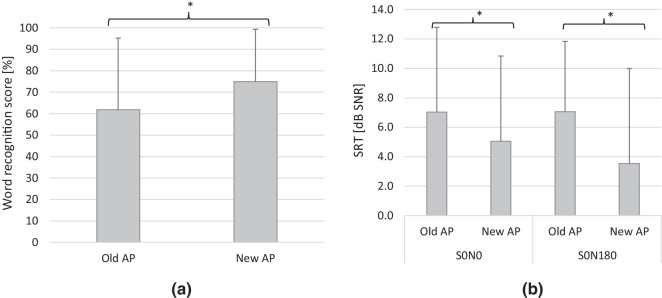
**a** Mean word recognition scores (WRS) in quiet in old and new AP; the bars are mean scores; the error bars represent standard deviation, *p < *0.05*. **b** Results of the Polish Matrix Sentence Test, Speech Recognition Thresholds (SRT) in old and new AP for different loudspeaker configurations, *S *signal, *N* noise, *0, 180* angles of incidence of sound; the bars are mean scores; the error bars represent standard deviation, *p < 0.05

The mean word recognition score in the unaided condition was 13.2% (SD = 25.4). The mean aided word recognition scores in quiet for old AP was 61.8% (SD = 33.4) whereas for new AP was 75.0% (SD = 24.3) (Fig. 1a). The difference of 13.2 percentage points in favor of the new AP was significant (*t*(21) = 3.92; *p* = 0.0008). A similar significant improvement of speech discrimination in noise was observed; mean results of the Polish Matrix Sentence Test using different loudspeaker configurations (S0-N0 and S0-N180) for the old and new AP are shown in Fig. 1b. In the S0-N0 configuration, SRT improved by 1.98 dB SNR (SD = 2.89; *t*(21) = 3.21; *p* = 0.004) when the new AP was compared to the old. In the S0-N180 configuration, the advantage of the new AP over the previous one was 3.52 dB SNR (SD = 3.83; *t*(21) = 4.30; *p* = 0.0003).

### Patient-Reported Outcome Measures

Evaluation with the SSQ questionnaire revealed that the post-upgrade Total score increased by 1.47 points over the pre-upgrade outcomes and this difference was significant. Similarly, significant increases were observed for Speech hearing (1.47), Spatial hearing (1.23), and Qualities of hearing (1.66). The mean results for the SSQ questionnaire and the results of pairwise comparisons are provided in Table 3.

**Table 3:** Results of PROMs

	Mean	SD	*t* test	*p* value
**SSQ**				
Total score				
Old AP	4.80	1.57	5.03	0.0001
New AP	6.27	1.57		
Speech hearing				
Old AP	4.36	2.09	3.87	0.0011
New AP	5.83	2.07		
Spatial hearing				
Old AP	5.03	1.73	3.31	0.0039
New AP	6.26	2.13		
Qualities of hearing				
Old AP	5.18	1.82	3.35	0.0035
New AP	6.84	1.32		
**APSQ**				
Total score				
Old AP	8.39	1.15	1.73	0.1007
New AP	8.80	0.89		
Comfort				
Old AP	8.20	1.09	0.10	0.9179
New AP	8.22	1.35		
Social life				
Old AP	8.26	1.73	1.54	0.1396
New AP	8.80	1.11		
Usability				
Old AP	8.76	1.31	2.12	0.0474
New AP	9.39	0.63		

*SD* standard deviation

For the APSQ questionnaire, the mean post-upgrade score, compared to pre-upgrade, increased by 0.41 points for Total score, 0.02 for Comfort, 0.53 for Social life, and 0.63 for Usability. The increase in the Usability dimension was found significant. Mean pre- and post-upgrade APSQ results and pairwise comparisons are presented in Table 3.

### PROMs and outcomes relationships

**Fig. 2:**
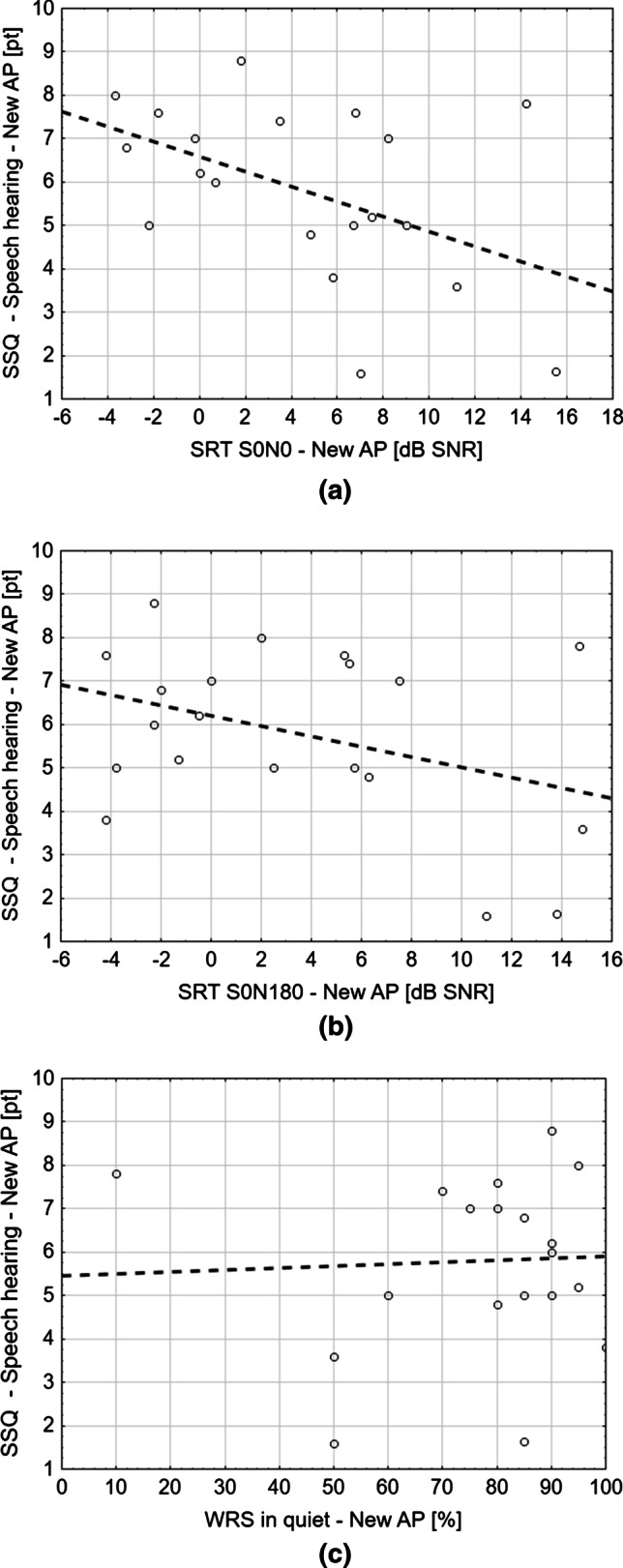
Correlation between Speech Hearing Score (SSQ) and results of speech tests with a new AP: **a** in noise S0-N0; **b** in noise S0-N180; **c** in quiet

The investigation of relationships between speech outcomes and PROM results revealed a negative correlation between speech discrimination in noise in the S0-N0 condition and the Speech hearing dimension of the SSQ questionnaire (*r* = − 0.48, *p* = 0.03). A similar relation (*r* = − 0.38), although not significant, was observed for speech discrimination in noise in the S0-N180 condition and the Speech hearing dimension. However, there was no correlation between speech discrimination in quiet and this dimension of SSQ (Fig. 2). In addition, we did not find any correlations between speech outcomes and SSQ or APSQ total score, or any other dimensions of those questionnaires.

## Discussion

To the authors' knowledge, there are few studies on upgrading the VSB audio processor. Older works compare the benefits of the 3-channel processor to the 8-channel, showing an auditory benefit [21, 22]. In the study of Todt et al. in 2005, the D-type audio processor was replaced by a Signia-type processor in three patients [22]. Due to the small number and markedly older technology of processors, the results are not comparable with those obtained here.

Table 4 gives an overview of recent studies. These studies cover speech discrimination in quiet and in noise, free-field thresholds, and PROMs. In all three studies there were no statistically significant differences between the old and the newer APs in terms of hearing thresholds in free-field. By way of contrast, in the study here there were statistically significant differences in mean hearing thresholds: in the old processor the mean PTA4 value was 42 dB HL, whereas in the new it was 38 dB HL.

**Table 4:** Recent studies that included assessment of the new technology in the Vibrant Soundbridge

Study	Subject	Audio processors	Speech in quiet	Speech in noise	Free-field thresholds	PROMs
Mühlmeier et al. [17]	*N* = 14 adults	Amadé → Samba	**WRS65** WRS Amadé = 65% WRS Samba = 63.6% – Difference not significant	1. **OLSA S0 N180** SRT Amadé = 5.9 dB SNR SRT Samba = 2.3 dB SNR – Significant difference 2. **OLSA S180 N0** SRT Amadé = 2.9 dB SNR SRT Samba = 1.1 dB SNR – Significant difference	Mean aided Amadé FF thresholds = 41.3 dB Mean aided Samba FF thresholds = 40.4 dB – Difference not significant	**APHAB** – Difference not significant **HDSS** – Difference not significant
Zimmermann et al. [19]	*N* = 20 adults	Amadé → Samba	**WRS65** WRS Amadé = 76% WRS Samba = 59% – Significant difference	1. **OLSA S0 Ncontra** **Samba vs Amadé** – Omni: significant advantage of 3.8 dB SNR for Samba – Directional: difference not significant 2. **OLSA S0 NVSB** **Samba vs Amadé** – Omni: significant advantage of 2.5 dB SNR for Samba – Directional: 1.1 dB SNR better with Samba, not significant	Mean aided Amadé FF thresholds = 38 dB Mean aided Samba FF thresholds = 38 dB – Difference not significant	**APHAB** – Significant difference in Background Noise subscale **SSQ-C** – Significant difference
Rahne et al. [12]	*N* = 15 adults	Samba → Samba 2	**WRS65** WRS Samba = 66% WRS Samba 2 = 74% – Significant difference	1. **OLSA Olnoise (S0, N120, 180, 240)** SRT Samba = − 5.4 dB SNR SRT Samba 2 = − 7.7 dB SNR – Significant difference 2. **OLSA ISTS (S0, N120, ISTS180, N240)** SRT Samba = − 4.8 dB SNR SRT Samba 2 = − 7.1 dB SNR – Significant difference	Mean aided Samba FF thresholds = 36.9 dB Mean aided Samba 2 FF thresholds = 36.7 dB – Difference not significant	**APSQ** Social life, Usability and Total Score – Significant difference **SSQ** – Significant difference

In terms of speech discrimination in quiet, only Rahne et al. showed a statistically significant difference in WRS in favor of the new processor, with an improvement from 66 to 74% when the Samba processor was changed to the Samba 2. These WRS scores are in line with the results of our study, where the WRS in the old processor (D404 or Amadé) was 61% compared to 75% with the new Samba 2.

The results of speech-in-noise discrimination from previous studies are more difficult to compare with our results. The reasons are methodological. Although all authors used adaptive tests, differences in loudspeaker configuration, sound direction, word material, processor settings, and other details were employed. In the work of Mühlmeier et al., the measurement conditions largely corresponded to those used in our study, although a constant noise of 70 dB SPL was used instead of the 65 dB SPL used in our work [23]. The SRT obtained for the S0-N180 condition was 5.9 dB SNR for the Amadé and 2.3 dB SNR for the Samba processor, a difference which was statistically significant [14]). For similar measurement conditions in our work, the SRT was 7.0 and 3.5 dB SNR for the old and new processors, respectively. In the case of replacing the processors with the latest Samba 2, Rahne et al. observed a statistically significant improvement in speech-in-noise discrimination after the use of a newer processor for multiple configurations of speech and noise sources. However, the types of stimuli and noise, as well as the spatial configurations of the loudspeakers, were different from what we used here. Nevertheless, the conditions used (S0-N120, N180, N240) are roughly similar to the one (S0-N180) used in our paper (since in both studies speech was presented from the front and noise from the back). Thus, we can broadly compare the improvement of 2.3 dB identified by Rahne et al. for S0-N120, N180, and N240 to the 3.5 dB improvement for the S0-N180 condition in our study. The slightly larger improvement reported by us could stem from the extra noise sources used by Rahne et al. (N120, N240) which could reduce the benefit from the directional microphone in the new processors.

Because speech and noise signals were spatially separated in our work, the differences in speech reception threshold in noise between the old and new processors may be due to a markedly difference in the way the microphones in the older and newer processors operate. The previous generation processors used an omnidirectional mode microphone, whereas the Samba 2 processor uses an advanced directional microphone system that is automatically adaptive.

In our study, a statistically significant difference was observed for the S0-N0 condition. A better result was obtained for the newer processor (Samba 2), with an SRT of 5.0 dB SNR compared to the 7.0 dB SNR for the older ones. The difference in SRT between APs was smaller when signal and noise were spatially separated.

There are certain difficulties in determining the most appropriate measurement setup for assessing modern technologies—for example, directional microphones, speech tracking, acoustic scene analysis, and others. The results of tests for hearing sensitivity and speech discrimination obtained in a clinical setup may not correspond with patients' needs and expectations in everyday life. This is the reason questionnaire tools (PROMs) were also used.

There are two other reasons for using PROMs. First, it is not easy to test new front-end processing features. During a single test session, simulating multiple environments is difficult, and so the user’s self-report (the PROM) becomes an important measure of how well this new technology works under different common situations. Second, there are many real-life situations—such as activity limitations and participation restrictions—which cannot be gauged by a speech discrimination test. These problems are unique and depend on personal circumstances, family situation, life-style, and so on, making PROMs necessary to quantify performance [24–26].

In the current study, the PROM results pointed to an appreciable subjective improvement when the speech processor was upgraded to the new technology. According to SSQ, patients reported less hearing disability and more satisfaction with the new AP, particularly in terms of its usability (APSQ).

Patients indicated that, compared to the legacy processor, the Samba 2 gave better spatial hearing, speech hearing, and better other qualities of hearing. Spatial hearing involves judgements of direction, distance, and movement. Speech hearing relates to diverse situations: noisy background conditions, reverberation, multiple voices, and the ability to ignore one voice while attending to another, following a conversation that switches quickly from one person to another, or following two speakers simultaneously. Other qualities of hearing refer to signal segregation, identification/recognition, clarity, naturalness, and ease of listening [27]. Improvements in spatial hearing and in speech hearing due to the upgrade is especially encouraging, as it indicates clear advantages of the new technologies, particularly automatic scene analysis when listening in difficult acoustic conditions.

In two previous studies in which the processor was replaced with a newer one, the SSQ questionnaire was used, after which a statistically significant improvement was noticed on all three dimensions: speech hearing, spatial hearing, and qualities of hearing [18, 28].

In the work of Zimmermann et al., the improvements with the new processor in the speech hearing dimension were on average 1.1 points, in the work of Rahne et al. they were 2.0 points, and in our study 1.5. In the spatial hearing dimension, the improvements were 0.8 points in Zimmermann's work, 1.7 in Rahne's, and 1.2 in ours. In the qualities of hearing dimension, the improvement reported by Zimmermann was 1.7 points, by Rahne et al. 1.5, and in our study 1.7. The average SSQ total score calculated in Rahne et al.'s work was 5.2 in the old processor and 7.0 in the new, while in our work it was 4.8 in the old and 6.3 in the new. In general, the differences between SSQ scores obtained with the old and new processors were statistically significant, both for the total score and for the individual dimensions, and consistent with the ones reported in the literature.

In the APSQ, the results obtained for both the new and old AP for each of the three dimensions exceeded 8 points. A statistically significant difference between the results for the new and old processors was obtained only for the usability dimension (9.4 for the new vs. 8.8 for the old). This dimension consists of questions about the ease of placing the AP properly on the head, ease of changing the battery, ease of switching on and off, proper functioning of the AP, and ease of care. For the APSQ total score, the difference was not statistically significant. In the work of Rahne et al., the mean total score was 8.2 for the older and 9.0 points for newer processors. They observed statistically significant improvements in total score, social life, and usability. In the usability dimension, the difference between the older and newer processor was 0.8 points, compared to 0.6 in our study.

As already stated, in the patient-centered care model, it is important not only for clinicians to obtain objective, measurable benefits under experimental conditions, but also that patients themselves report benefits in everyday functioning. The rationale is that numerous publications have shown that audiological measures generally correlate poorly with PROMs [29–32]. Dornhofer et al., in a group of 95 hearing aid users, correlated aided audiological measures (PTA, Nu-6, SPIN) with aided APHAB subscales and global score; they saw no significant relationship. Absent or low correlations between patient self-reported scores and speech recognition have also been reported among cochlear implant users [33–35]. Mertens et al. reported that self-assessment tools, like SSQ, offer insights into dynamic hearing capacities that cannot be easily measured in the laboratory and provide useful information about the hearing status of CI users [33]. The results obtained in this study are consistent with the conclusions from other work, confirming the lack of correlation (or weak correlation) between the results of speech discrimination in quiet and noise and the results of PROMs. When testing the relationship between speech hearing dimension in the SSQ and the result of speech tests with the new AP, a significant negative correlation was obtained for S0-N0; a smaller (also negative) but not significant correlation for S0-N180, and no correlation for WRS obtained in quiet. A similar relationship was noted by Remakers et al., who found, using SSQ, a significant (but weak to moderate) negative correlation between the subjective test results of the speech hearing dimension and the related objective speech perception in noise test [35]. In the recently published ‘Consensus Statement on Bone Conduction Devices and Active Middle Ear Implants in Conductive and Mixed Hearing Loss’, the authors address an important issue, noting that companies introducing new processors should enable patients with older implants to reap the benefits of new features and signal processing developments [36]. We see the opportunity here for long-term users of implantable devices: they do not need surgical intervention to gain access to modern technologies (ignoring, of course, issues relating to cost and insurance). Upgrades offer a way of reducing the impact of hearing impairment in everyday life and offering better functional performance.

## Conclusion

By replacing the processor, modern technology can bring measurable benefits, particularly in terms of speech discrimination. PROMs confirm the obtained benefits: a reduced impact of hearing impairment on everyday functioning. Patients also notice the practical, functional benefits of the newer solutions. This study has shown that the use of the latest solutions—adaptive microphone directionality, speech tracking, and environmental sound classification—can provide significant benefits to patients.

## Data Availability

The datasets used and/or analysed during the current study are available from the corresponding author on reasonable request.
